# [Corrigendum] Reactive oxygen species-mediated activation of the Src-epidermal growth factor receptor-Akt signaling cascade prevents bortezomib-induced apoptosis in hepatocellular carcinoma cells

**DOI:** 10.3892/mmr.2025.13677

**Published:** 2025-09-08

**Authors:** Jinlin Hou, Anguo Cui, Peiying Song, Hui Hua, Ting Luo, Yangfu Jiang

Mol Med Rep 11: 712–718, 2015; DOI: 10.3892/mmr.2014.2736

Following the publication of the above article, the authors have realized that there were several duplicated western blots featured in Fig. 2, Fig. 3, Fig. 4. The EGFR blot correctly presented for Fig. 3 (the HepG2 cell line) had been inadvertently featured in Fig. 2 (the EGFR blot for the HepG2 cell line). In Fig. 3, the incorrectly assembled Akt blot for the SMMC-7721 group was the source image for the correctly presented Akt band in Fig. 1 (the SMMC-7721 cell line), whereas the Akt blot correctly presented for the Hep3B cell line had been inadvertently featured as the actin blot of the HepG2 cell line. The p-Src blot correctly presented for Fig. 2 (the SMMC-7721 cell line) had been inadvertently featured in Fig. 4C (the p-Src blot for SMMC-7721). Additionally, the actin blots correctly presented for Fig. 2 (the SMMC-7721 and HepG2 cell lines) were incorrectly assembled into Fig. 4C (the actin blots for SMMC-7721 and HepG2).

The revised versions of Fig. 2, Fig. 3, Fig. 4, now showing the correct data for the EGFR blot of the HepG2 cell line in Fig. 2, the Akt blot for the SMMC-7721 cell line and the actin blot for the HepG2 cell line in Fig. 3, the p-Src blot for the SMMC-7721 cell line and the actin blots for SMMC 7721 and HepG2 in Fig. 4C, are shown on the next two pages. The authors wish to state that these errors were made during the figure assembly process, and did not affect either the results or the conclusions reported in this paper. All the authors agree with the publication of this corrigendum, and are grateful to the Editor of *Molecular Medicine Reports* for granting them the opportunity to publish this. The authors regret that these errors were included in the paper, and also apologize to the readership for any inconvenience caused.

## Figures and Tables

**Figure 2.**
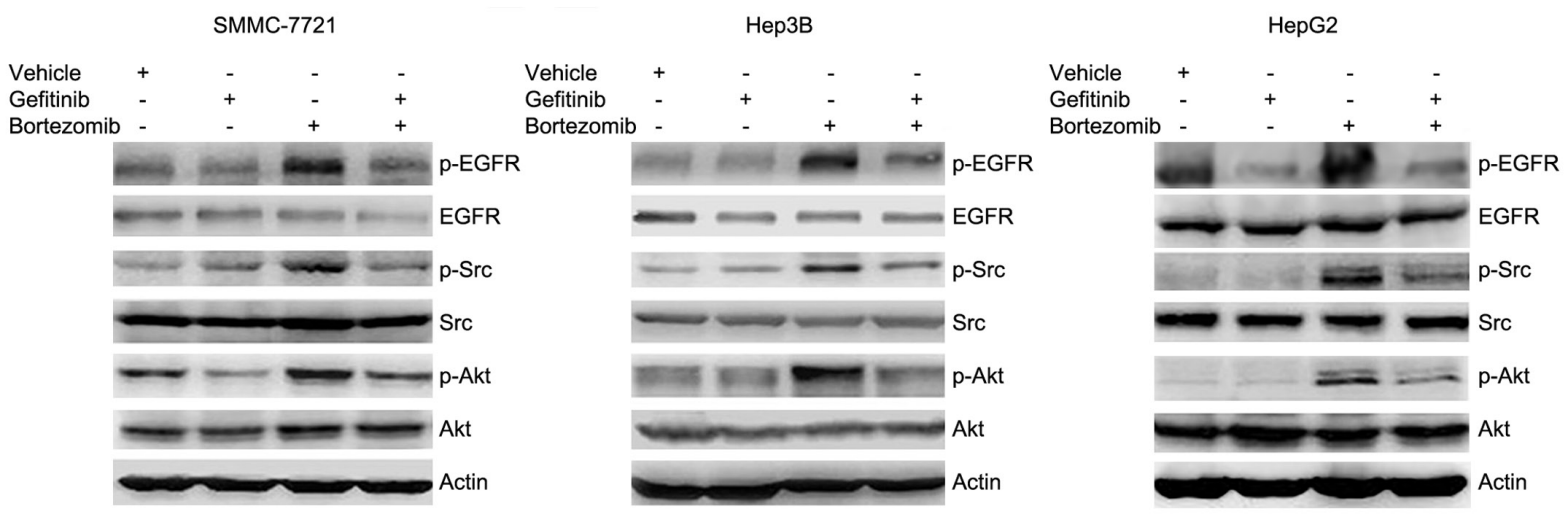
Gefitinib inhibits bortezomib-induced EGFR, Src and Akt phosphorylation. SMMC-7721, Hep3B and HepG2 cells were treated with or without 10 μM gefitinib and 100 nM bortezomib for 24 h, followed by western blot analysis of EGFR, Src and Akt phosphorylation. EGFR, epidermal growth factor receptor; p-, phosphorylated.

**Figure 3.**
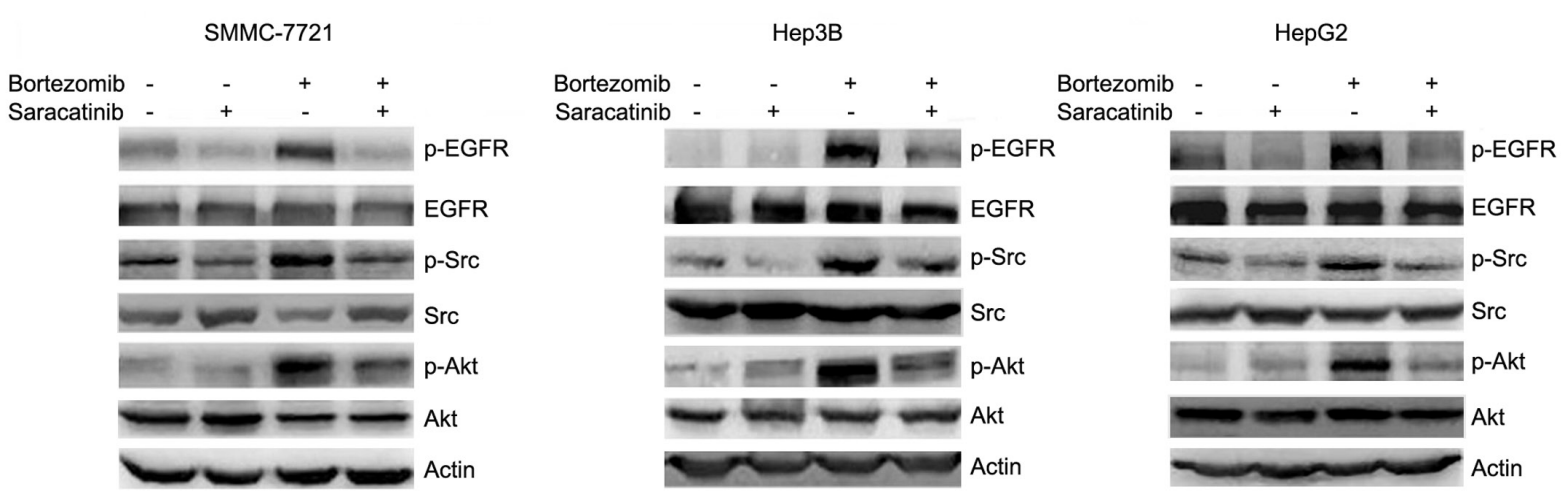
Saracatinib decreases bortezomib-induced EGFR, Src and Akt phosphorylation. SMMC-7721, Hep3B and HepG2 cells were treated with or without 1.5 μM saracatinib and 100 nM bortezomib for 24 h, followed by western blot analysis of EGFR, Src and Akt phosphorylation. EGFR, epidermal growth factor receptor; p-, phosphorylated..

**Figure 4.**
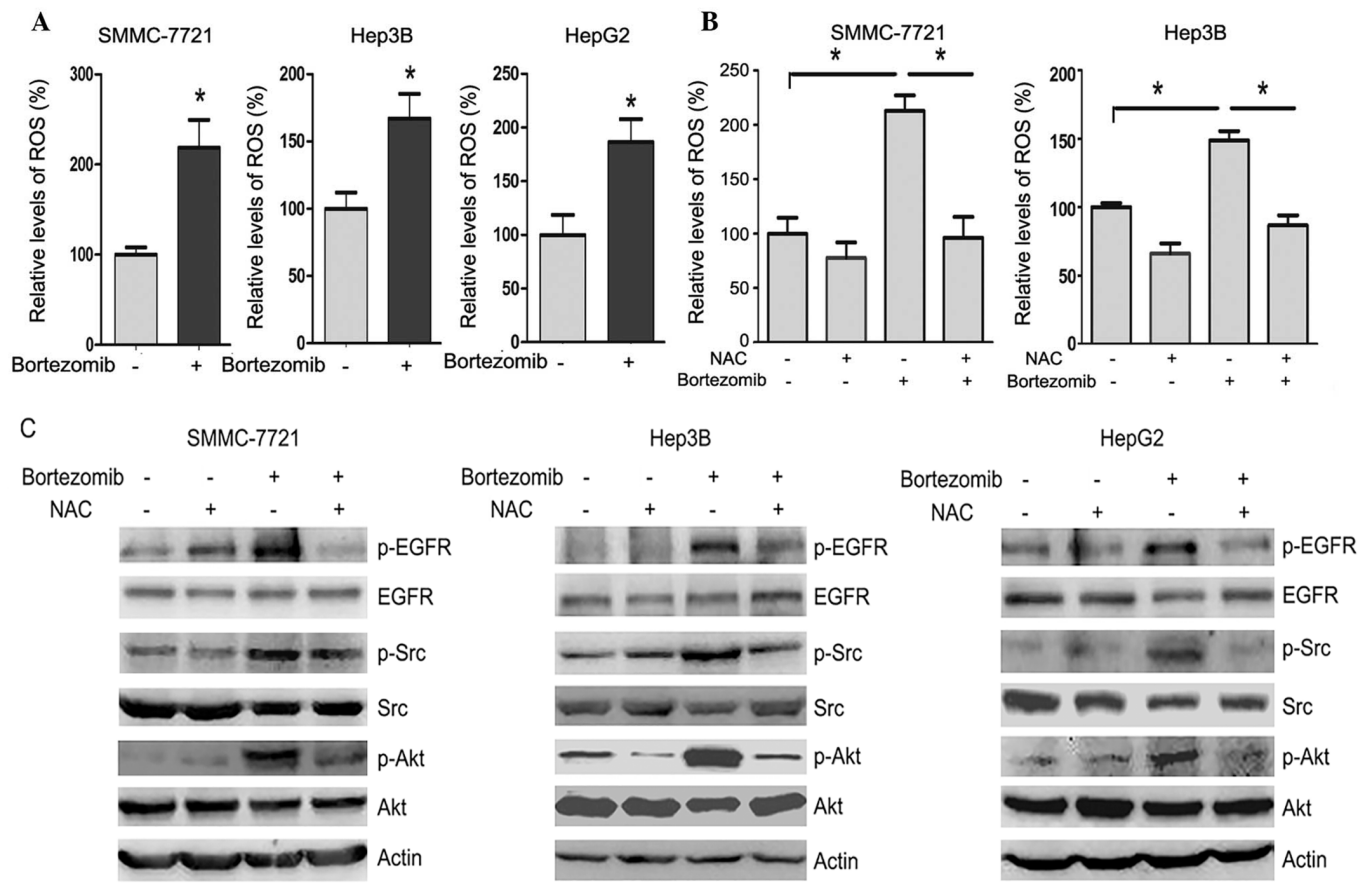
ROS mediate the induction of Src, EGFR and Akt phosphorylation by bortezomib. (A) SMMC-7721, Hep3B and HepG2 cells treated with or without 100 nM bortezomib for 24 h, followed by detection of intracellular ROS. The relative levels of ROS were plotted. Values are expressed as the mean ± SD. *P<0.05 vs. control. (B) SMMC-7721 and Hep3B cells treated with or without 5 mM NAC and 100 nM bortezomib for 24 h, followed by detection of intracellular ROS. The relative levels of ROS were plotted. Values are expressed as the mean ± SD. *P<0.05. (C) SMMC-7721, Hep3B and HepG2 cells treated with or without 5 mM NAC and 100 nM bortezomib for 24 h, followed by western blot analysis of EGFR, Src and Akt phosphorylation. ROS, reactive oxygen species; EGFR, epidermal growth factor receptor; p-, phosphorylated; SD, standard deviation; NAC, *N*-acetyl cysteine..

